# Inhibition effects of patchouli alcohol against influenza a virus through targeting cellular PI3K/Akt and ERK/MAPK signaling pathways

**DOI:** 10.1186/s12985-019-1266-x

**Published:** 2019-12-23

**Authors:** Yunjia Yu, Yang Zhang, Shuyao Wang, Wei Liu, Cui Hao, Wei Wang

**Affiliations:** 1 Key Laboratory of Marine Drugs, Ministry of Education, Ocean University of China, Qingdao, 266003 People’s Republic of China; 2 Systems Biology & Medicine Center for Complex Diseases, Affiliated Hospital of Qingdao University, Qingdao, 266003 People’s Republic of China; 3 Laboratory for Marine Drugs and Bioproducts of Qingdao National Laboratory for Marine Science and Technology, Qingdao, 266237 People’s Republic of China

**Keywords:** Patchouli alcohol, Influenza a virus, Antiviral effects, PI3K/Akt pathway, ERK/MAPK pathway

## Abstract

**Background:**

Patchouli alcohol (PA) is a tricyclic sesquiterpene extracted from *Pogostemonis Herba*, which is a traditional Chinese medicine used for therapy of inflammatory diseases. Recent studies have shown that PA has various pharmacological activities, including anti-bacterial and anti-viral effects.

**Methods:**

In this study, the anti-influenza virus (IAV) activities and mechanisms were investigated both in vitro and in vivo. The inhibitory effects of PA against IAV in vitro were evaluated by plaque assay and immunofluorescence assay. The neuraminidase inhibition assay, hemagglutination inhibition (HI) assay, and western blot assay were used to explore the anti-viral mechanisms. The anti-IAV activities in vivo were determined by mice pneumonia model and HE staining.

**Results:**

The results showed that PA significantly inhibited different IAV strains multiplication in vitro, and may block IAV infection through inactivating virus particles directly and interfering with some early stages after virus adsorption. Cellular PI3K/Akt and ERK/MAPK signaling pathways may be involved in the anti-IAV actions of PA. Intranasal administration of PA markedly improved mice survival and attenuated pneumonia symptoms in IAV infected mice, comparable to the effects of Oseltamivir.

**Conclusions:**

Therefore, Patchouli alcohol has the potential to be developed into a novel anti-IAV agent in the future.

## Background

Influenza A Virus (IAV) belongs to the *Orthomyxoviridae* family, being segmented, single stranded, negative sense RNA viruses [1]. IAV caused at least three large-scale influenza outbreaks in the twentieth century, the most serious of which was the Spanish influenza outbreak in 1918, which caused more than 40 million deaths [2, 3]. More recently, the emergence and global spread of H1N1 influenza from the 2009 pandemic and recent lethal cases of H5N1 and H7N9 influenza demonstrate the limitations of currently available strategies to control influenza infection [4]. Currently, there are three main types of anti-IAV drugs approved for clinical use: (1) M2 ion channel inhibitors such as amantadine and rimantadine; (2) Neuraminidase inhibitors such as oseltamivir, zanamivir, and peramivir [5, 6]; (3) Polymerase inhibitors such as baloxavir. However, the emergence of drug-resistant influenza variants such as amantadine and oseltamivir resistant IAV strains has led to a decline in the efficacy of these drugs. In addition, most of these anti-IAV drugs also have some side effects such as nervous system damage [7–9]. Therefore, new influenza therapeutics with novel mechanisms of action are urgently required to combat the persistent threat of influenza viruses.

Patchouli alcohol is a tricyclic sesquiterpene extracted from *Pogostemonis Herba*, which has long been used in the treatment of inflammatory diseases as a traditional Chinese medicine [10]. Recent studies have shown that patchouli oil has various pharmacological activities, including anti-emetic [11], anti-inflammatory [12], anti-bacterial [13], and anti-viral effects [14]. Li et al. found that oral administration of PA appeared to be able to augment protection against influenza virus infection in mice via enhancement of host immune responses, and attenuation of systemic and pulmonary inflammatory responses [15]. Wu and co-workers reported that patchouli alcohol inhibited influenza A (H2N2) virus mainly through interfering with the functions of virus neuraminidase [16]. Therefore, PA has the potential to be developed into a novel anti-viral agent in the future.

To further correlate the potential anti-IAV applications of PA with its underlying molecular mechanisms, the anti-IAV (H1N1) effects and mechanisms of PA were investigated in vitro and in vivo in this study. The results showed that PA may block IAV infection through inactivating IAV directly and interfering with some early steps after virus adsorption. Cellular PI3K/Akt and ERK/MAPK signaling pathways may be involved in the anti-IAV actions of PA. In addition, intranasal administration of PA markedly improved mice survival and attenuated pneumonia symptoms in IAV infected mice.

## Materials and methods

### Reagents

Patchouli alcohol (PA) (with purity > 98%) was purchased from TargetMol (Shanghai, China). Dulbecco’s Modified Eagle’s medium (DMEM), penicillin, and streptomycin were purchased from Gibco (Grand Island, NY, USA). Fetal bovine serum (FBS) was obtained from Excell (Suzhou, China). Mouse anti-influenza A virus NP antibody and alkaline phosphatase (AP)-labeled secondary antibodies were obtained from Santa Cruz Biotechnology (USA). DyLight 649 conjugated secondary antibody was obtained from Abbkine (California, USA). Ribavirin injection (50 mg/ml) was purchased from LuKang Cisen (Jining, China). Oseltamivir carboxylate was purchased from Santa Cruz Biotechnology (Santa Cruz, CA, USA). Oseltamivir phosphate was obtained from Roche (Shanghai, China). The influenza neuraminidase inhibitor detection kit was purchased from Beyotime (Shanghai, China). The anti-NP protein was provided by Abcam (ab128193), and other antibody, such as anti-phosphorylated PI3K(4228 s), Akt (9271 s), mTOR (5536 s), ERK1/2 (4370 s), and NF-κB (3033 s) antibodies, or anti-GAPDH (2118 s) and α-tubulin antibodies (2125 s), were obtained from Cell Signaling Technology (Danvers, USA).

### Cells and virus

Madin-Darby canine kidney (MDCK) cells were grown in DMEM medium supplemented with 10% FBS, 100 U/mL of penicillin and 100 μg/ml of streptomycin. A549 cells were cultivated in F12 medium containing 10% FBS and 2 mM L-glutamine. Influenza A virus H1N1 (A/Puerto Rico/8/34), H1N1 (A/NWS/33), and H1N1 (A/Virginia/ATCC1/2009) were propagated in 10-day-old embryonated eggs for 3 days at 36.5 °C. For infection, virus propagation solution was diluted in PBS containing 0.2% bovine serum albumin (BSA) and was added to cells at the indicated multiplicity of infection (MOI). Virus was allowed to adsorb 60 min at 37 °C. After removing the virus inoculum, cells were maintained in infecting media (DMEM, 4 μg/ml trypsin) at 37 °C in 5% CO_2_.

### Cytotoxicity assays

The cytotoxicity of compounds was measured by the MTT (Sigma–Aldrich, USA) assay. Confluent MDCK, A549 and 293FT cell cultures in 96-well plates were exposed to different concentrations of PA (6.25, 12.5, 25, 50, 100 μg/ml) in triplicate for 24 h. After that, 10 uL of PBS containing MTT (final concentration: 0.5 mg/mL) was added to each well. After 4 h incubation at 37 °C, the supernatant was removed and 200 uL of DMSO was added to each well to solubilize the formazan crystals. After vigorous shaking, absorbance values were measured in a microplate reader (Bio-Rad, USA) at 570 nm. The CC_50_ was calculated as the compound concentration necessary to reduce cell viability by 50%.

### Plaque reduction assay

Different concentrations (50, 25, 12.5, 6.25, 3.125 or 1.56 μg/mL) of PA in 500 μL DMEM media were mixed with an equal volume of infectious IAV (50–100 PFU/well) in DMEM, and incubated at 37 °C for 1 h. The virus-PA mixtures were then transferred to confluent MDCK cell monolayers in 12-well plates, and incubated at 37 °C for 1 h with gentle shaking every 15 min. After that, the inoculum was removed and each well was overlaid with 1 mL of agar overlay medium (1.5% agarose, 0.02% DEAE dextran, 1 mM L-glutamine, 0.1 mM non-essential amino acids, 100 U/ml penicillin, 100 mg/ml streptomycin and 1 mg/ml TPCK treated trypsin). After incubation for 3 days at 37 °C in 5% CO_2_, cells were fixed with 4% para-formaldehyde (PFA), followed by staining with 1% crystal violet for plaque counting.

### Time of addition study

MDCK cells were infected with H1N1 (Vir09, MOI =1.0) under four different treatment conditions. i) Pretreatment of virus: IAV was pretreated with 25 μg/ml of PA at 37 °C for 1 h before infection. ii) Pretreatment of cells: MDCK cells were pretreated with 25 μg/ml of PA at 37 °C for 1 h before infection. iii) Adsorption: MDCK cells were infected in media containing 25 μg/ml of PA and, after 1 h adsorption at 37 °C, were overlaid with compound-free media. iv) After adsorption: After 1 h adsorption at 37 °C, the inoculum was removed and the infecting media containing 25 μg/ml of PA were added to cells. At 24 h p.i., the antiviral activity was determined by plaque assay. Mean percentage virus titers were calculated as a percentage of plaque titers from untreated control group.

### Indirect immunofluorescence assay

IAV virus (MOI = 3.0) infected A549 cells were treated with or without PA (10, 20 μg/ml) after adsorption. At 2 h post infection, MDCK cells were fixed with 4% PFA for 15 min. Then cells were permeabilized and incubated sequentially with primary antibodies against IAV NP protein and DyLight 649 conjugated secondary antibody. Then after washing, the cell nucleus was stained with DAPI for 20 min before confocal imaging. Images were recorded using a Nikon confocal microscope, and analyzed by ImageJ (NIH) version 1.33 u (USA).

### Hemagglutination (HA) assay

The hemagglutination (HA) assay was performed as previously reported [17]. Standardized chicken red blood cell (cRBC) solutions were prepared according to the WHO manual. Virus propagation solutions were serially diluted 2-fold in round bottomed 96-well plate and 1% cRBCs were then added at an equal volume. After 60 min incubation at 4 °C, RBCs in negative wells sedimented and formed red buttons, whereas positive wells had an opaque appearance with no sedimentation.

### Mini-genome assay

293FT cells were cultured in 24 well plates at 37 °C for 24 h before transfected with plasmids (PB2/pcDNA3.1, PB1/pcDNA3.1, PA/pcDNA3.1, or NP/pcDNA3.1) encoding PR8 virus polymerase subunits (PA, PB1, and PB2 protein) and virus nucleoprotein (NP), and a luciferase RNA expression vector (vNS1-luc/pHH21). Then the cells were treated with or without PA (3.125, 6.25, 12.5, 25 and 50 μg/ml) or Nucleozin (10 μM). The effect of vRNA transcription was then evaluated by measuring luciferase activity according to manufacturer’s instructions after incubation at 37 °C for 48 h.

### Neuraminidase inhibition assay

The influenza neuraminidase inhibitor detection kit was used to measure the inhibition of NA activity [17]. Briefly, inactivated PR8 virus supernatants was added to a 96-well plate and then mixed with different compounds (diluted in 33 mM MES buffer (pH 3.5), 4 mM CaCl_2_) at 37 °C for 30 min. Then MUNANA (20 μM) was added as the substrate and incubated at 37 °C for 30 min. The reaction was stopped by the addition of stop solution (25% ethanol, 0.1 M glycine, pH 10.7). Fluorescence was measured using a SpectraMax M5 plate reader with excitation and emission wavelengths of 360 and 440 nm, respectively.

### Quantitative RT-PCR assay

Total RNA was extracted from Vir09 virus (MOI = 1.0) infected MDCK cells using an RNAiso™ Plus Kit (Takara, Japan), and analysed by using the One Step SYBR PrimeScript RT-PCR Kit (Takara, Japan). The real-time RT-PCR was performed using the following primers: virus HA mRNA, 5′-AAGTCCTCGTGCTATGGG-3′ and 5′-TGGGAGGCTGGTGTTTAT-3′; β-actin mRNA, 5′-CTCCATCCTGGCCTCGCTGT-3′ and 5′-GCTGTCACCTTCACCGTTCC-3′. The real-time RT-PCR was performed at 42 °C 5 min, 95 °C 10 s, 40 cycles of 95 °C 5 s, 60 °C 34 s, followed by melting curve analysis, according to the instrument documentation (ABI PRISM 7500, Applied Biosystems, USA). The relative amounts of virus HA mRNA molecules were determined using the comparative (2^-ΔΔCT^) method, as previously described (18).

### Western blot assay

After drug treatment, the cell lysate was separated by SDS-PAGE and transferred to nitrocellulose membrane. After being blocked in Tris-buffered saline (TBS) containing 0.1% Tween 20 (v/v) and 5% BSA (w/v) at room temperature for 2 h, the membranes were rinsed and incubated at 4 °C overnight with anti-NP protein (Santa Cruz, USA), anti-phosphorylated PI3K, Akt, ERK1/2, and NF-κB antibodies, or anti-GAPDH, β-actin, and α-tubulin antibodies (Cell Signaling Technology, Danvers, USA) as control. The membranes were washed and incubated with AP-labeled secondary antibody (1:2000 dilutions) at RT for 2 h. The protein bands were then visualized by incubating with the developing solution (p-nitro blue tetrazolium chloride (NBT) and 5-bromo-4-chloro-3-indolyl phosphate toluidine (BCIP) at RT for 30 min. The relative densities of proteins were all determined by using ImageJ (NIH) v.1.33 u (USA).

### Mice experiments

Four-week-old female Kunming mice (average weight, 14.0 ± 1.0 g) were housed and studied under protocols approved by the Animal Care and Use Committee of Ocean University of China (OUCYY-2018003). Mice received humane care in accordance with the guidelines provided by the National Institutes of Health for the use of animals in laboratory experiments. 10 mice per group were inoculated intranasally with PR8 (2LD_50_/mouse) diluted in 40 uL of 1× PBS. All the mice were randomly divided into experimental groups. Four hours after inoculation, mice received intranasal therapy of PA (20 or 40 μg/day) or oral therapy of Oseltamivir phosphate (10 mg/kg/day), and the treatments were repeated once daily for 7 days. Mice were weighed and killed on day 4 after inoculation, and the lungs were then removed, weighed, and homogenized in 1 × PBS for determination of viral titers by plaque assay. Histopathological analysis was performed using H&E staining on samples collected on 4 days post infection (dpi) as described previously [18].

In the survival experiments, 10 mice per group were intranasally infected with PR/8 virus (4 LD_50_/mouse) at Day 0. The drugs administration was repeated once daily during the experiment, and survival was assessed in all groups for 14 days after infection. Mice were monitored daily for weight loss and clinical signs. If a mouse lost body weight over 25% of its pre-infection weight, it was defined as dead and humanely euthanized immediately; the rest of the mice were sacrificed at the end of experiment on 14 dpi.

### Statistical analysis

All data are representative of at least three independent experiments. Data are presented as means ± standard deviations (SD). Statistical significance was calculated by GraphPad Prism 7 software using one-way ANOVA with Turkey’s test, with *P* values < 0.05 considered significant.

## Results

### Inhibition of influenza a virus multiplication in vitro by patchouli alcohol

**Fig. 1:**
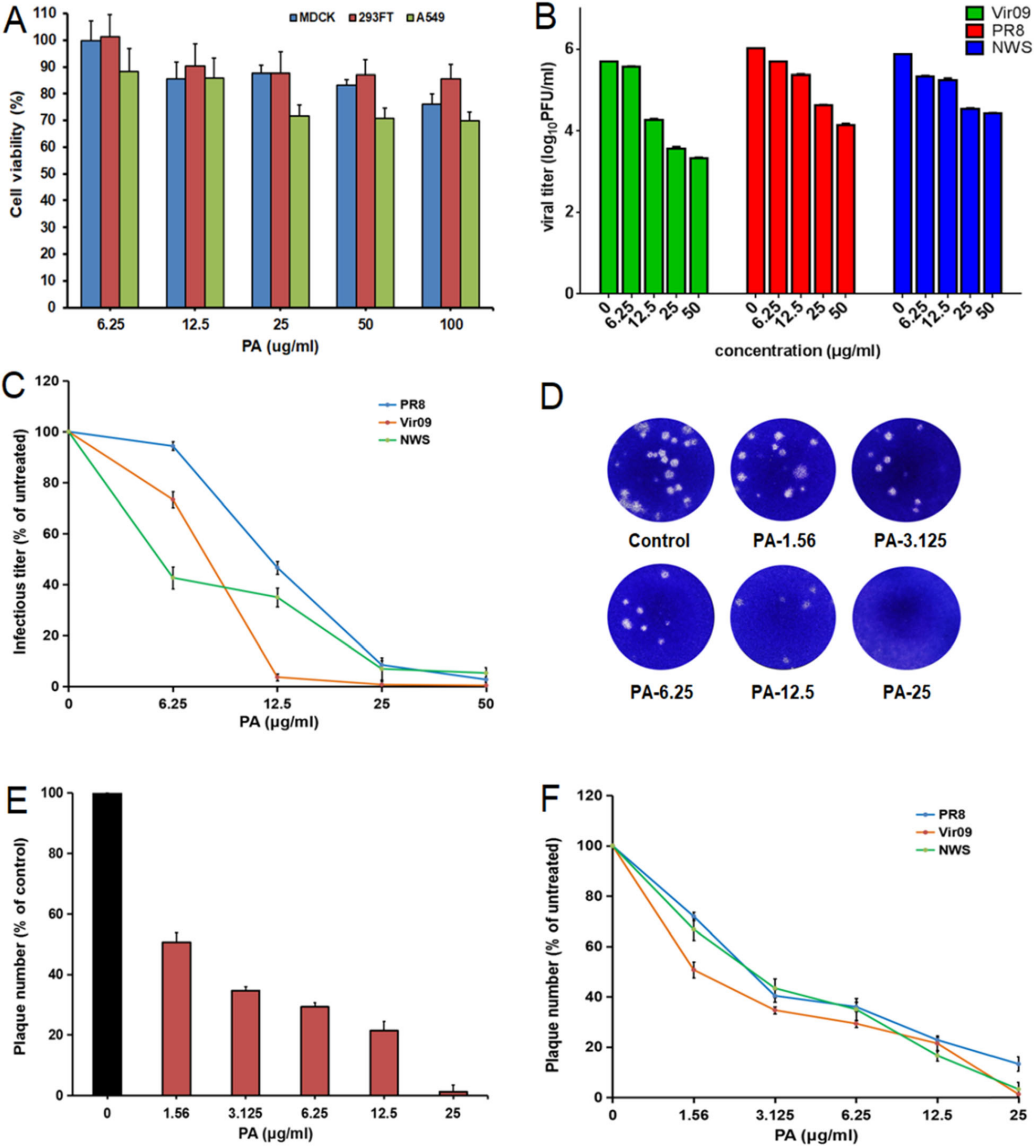
Patchouli alcohol inhibited replication of IAV in vitro with low toxicity. **a** After 24 h exposure to Patchouli alcohol (100, 50, 25, 12.5, 6.25 μg/ml) in MDCK, A549 and 293FT cells, the cell viability of Vero cells was measured by MTT assay. Values are means ± S.D. (*n* = 3). **b** IAV (MOI = 1.0) infected MDCK cells were treated with PA at the indicated concentrations for 24 h, then the antiviral activity was determined by plaque assay. Values are means±S.D. (*n* = 3). **c** Infectious virus titers from single-cycle high-moi assays performed on MDCK cells infected with PR8, Vir09 and NWS and treated with the indicated concentrations of PA. Mean percentage infectious virus titers were calculated as a percentage of infectious virus titers from untreated cells for each drug treatment condition in an experiment. Values are means ± S.D. (*n* = 3). **d** Approximately 50–100 PFU/well of Vir09 virus was pre-incubated with different concentrations of PA for 60 min at 37 °C before infection. Then the virus-PA mixture was transferred to confluent cell monolayers in 6-well plates, incubated at 37 °C for 1 h and subjected to plaque assay. **e** Plaque number from plaque reduction assays performed on MDCK cells infected with Vir09 and treated with the indicated concentrations of PA. Values are means ± S.D. (*n* = 4). **f** Plaque number from plaque reduction assays performed on MDCK cells infected with PR8, Vir09 and NWS and treated with the indicated concentrations of PA. Mean percentage plaque numbers were calculated as a percentage of plaque numbers from untreated cells for each drug treatment condition in an experiment. Values are means ± S.D. (*n* = 3)

The cytotoxicity of Patchouli alcohol (PA) was firstly evaluated by MTT assay in MDCK, A549 and 293FT cells [19]. The results showed that PA exhibited no significant cytotoxicity at the concentrations from 6.25 to 100 μg/ml (Fig. 1a). The CC_50_ (50% Cytotoxicity Concentration) values for PA in MDCK, 293FT and A549 cells were about 550.8, 914.8, and 454.5 μg/ml, respectively. These results were used to determine the dose range of PA for the subsequent experiments.

PA was then assayed for its ability to inhibit IAV multiplication in vitro using plaque assay [20]. Firstly, the inhibition of PA on the virus yields from MDCK cells infected with Vir09 (A/Virginia/ATCC1/2009), NWS (A/NWS/33) or PR8 (A/Puerto Rico/8/34) at high moi (≈1.0 PFU/cell) were examined by plaque assay. As shown in Fig. 1b and c, PA treatment reduced the virus titers of Vir09, NWS, and PR8 in a dose-dependent manner when used at the concentrations of 6.25–50 μg/mL. The 50% inhibitory concentration (IC_50_ value) of PA for Vir09, NWS, and PR8 was about 6.3 ± 1.3, 3.5 ± 1.4, and 6.1 ± 1.7 μg/mL, respectively (Table 1). At the concentration of 12.5 μg/ml, the virus titers reduced about 30 fold of that in the untreated control group for Vir09, 3.0 fold of that for NWS, and 2.5 fold of that for PR8 virus (Fig. 1b and c).

**Table 1:** The inhibitory effects of PA against different IAV strains in vitro

Compound	Virus strains	Single-cycle replication assay^a^	Multicycle replication assay^a^
		Infectious virus titer	Plaque number
		IC_50_ (μg/ml)^b^	IC_50_ (μg/ml)^b^
PA	Vir09	6.3 ± 1.3	2.2 ± 0.2
	NWS	3.5 ± 1.4	3.2 ± 0.2
	PR8	6.1 ± 1.7	2.9 ± 0.4
Ribavirin	Vir09	0.1 ± 0.02	121.0 ± 22.1

^a^Single-cycle high-moi assays and multicycle plaque reduction assays were performed on MDCK cells infected with Vir09, NWS, and PR8. Values are means ± S.D. (*n* = 3)

^b^Inhibition concentration 50% (IC_50_): concentration required to reduce the virus titer or plaque number by 50%

To further explore whether PA had direct inhibition actions on viral particles, the plaque reduction assay was performed as previously described [21]. In brief, Vir09 virus (50–100 PFU/well) was pre-incubated with or without PA for 60 min at 37 °C before infection. Ten the virus-PA mixture was transferred to confluent cell monolayers in 6-well plates incubated at 37 °C for 1 h and subjected to plaque assay. As shown in Fig. 1d and e, pre-incubation of PR8 with PA at the concentrations of 12.5 and 25 μg/ml markedly reduced the number of plaques and protected MDCK cells, suggesting that PA may be able to inactivate viral particles directly.

Furthermore, the inhibition effects of PA on IAV infection was also examined over multiple cycles of infection using plaque reduction assay [17]. Briefly, MDCK cells were infected with PA pretreated virus (Vir09, NWS, and PR8) at an moi of 0.001 pfu for 1 h at 37 °C, and then subjected to plaque assay. As shown in Fig. 1f, PA also significantly inhibited the plaque formation in Vir09, NWS and PR8 (MOI = 0.001) infected cells when used at the concentration > 3.125 μg/ml (Fig. 1f). The IC_50_ values of PA for Vir09, NWS, and PR8 was about 2.2 ± 0.2, 3.2 ± 0.2, and 2.9 ± 0.4 μg/ml, respectively (Table 1). However, ribavirin could not significantly inhibit the plaque formation of Vir09 with IC_50_ value > 120 μg/ml (Table 1). Thus, PA possessed anti-IAV effects in vitro, and the pandemic H1N1 virus (Vir09) was most susceptible to PA treatment.

### Influence of different treatment conditions of PA on IAV infection

**Fig. 2:**
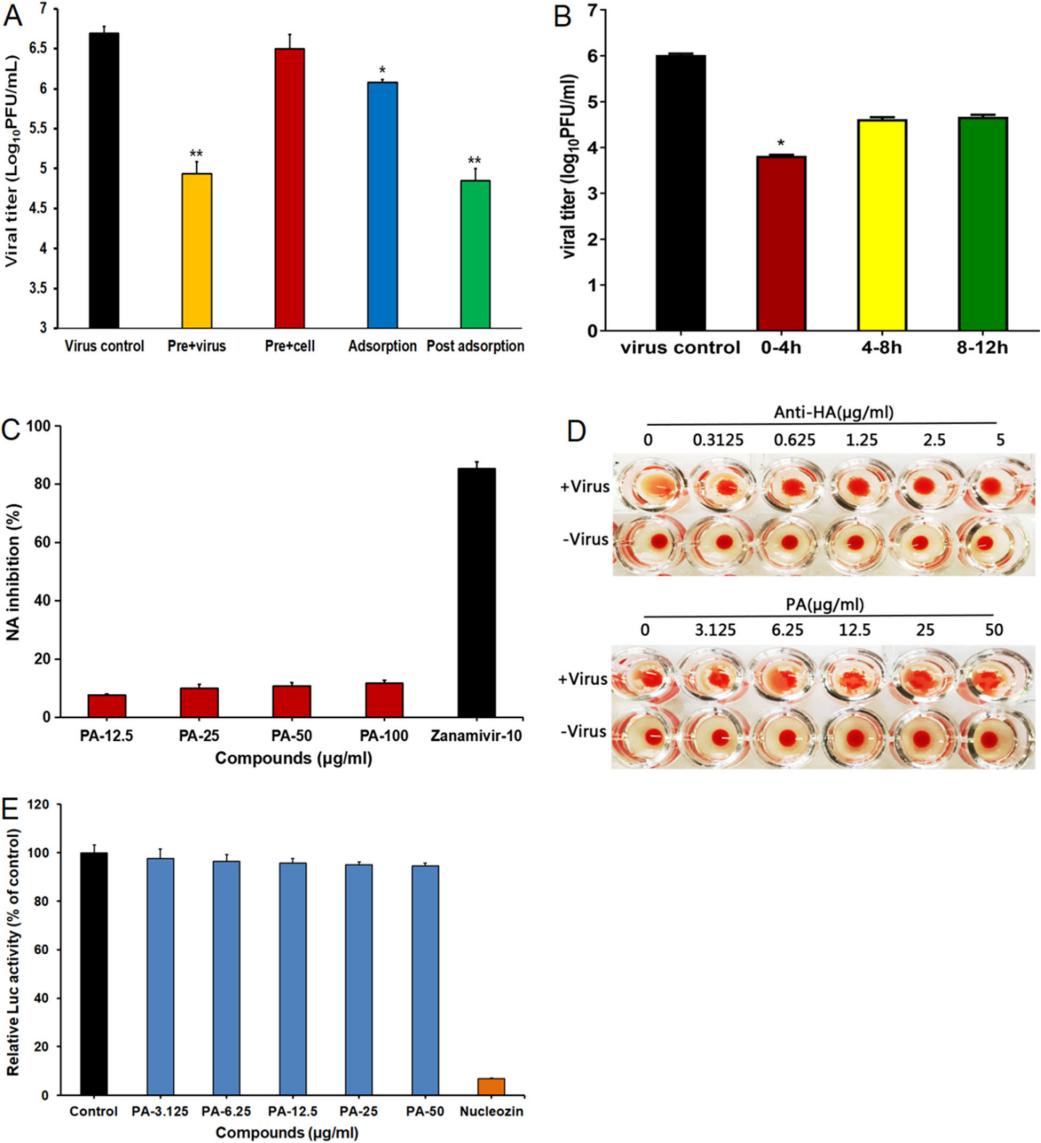
Influence of different treatment conditions of Patchouli alcohol on IAV infection. **a** MDCK cells were infected with H1N1 (Vir09, MOI =1.0) by four different treatment conditions. i) Pretreatment of virus: IAV was pretreated with 25 μg/ml of PA at 37 °C for 1 h before infection. ii) Pretreatment of cells: MDCK cells were pretreated with 25 μg/ml of PA at 37 °C for 1 h before infection. iii) Adsorption: MDCK cells were infected in media containing 25 μg/ml of PA and, after 1 h adsorption at 37 °C, were overlaid with compound-free media. iv) After adsorption: after removal of unabsorbed virus the infecting media containing 25 μg/ml of PA were added to cells. At 24 h p.i., the antiviral activity was determined by plaque assay. Values are means ± S.D. (*n* = 3). *P < 0.05, **P < 0.01 vs. virus control group. **b** PR8 (MOI = 1.0) infected MDCK cells were treated with or without 25 μg/ml of PA for the specified time period, and then the media were removed and cells were overlaid with compound-free media. Then at 24 h p.i., the cell supernatants were collected and the virus yields were determined by plaque assay. Values are means ± S.D. (*n* = 3). Significance: *p < 0.05 vs. virus control group. **c** Inactivated H1N1 (Vir09) virus and H1N1(PR8) was incubated with indicated concentrations of PA or Zanamivir (10 μg/ml), and the NA enzymatic activity was determined by a fluorescent assay. Values are means ± S.D. (*n* = 3). **d** The inhibition effects of PA and anti-HA antibody on influenza virus H1N1 (PR8) induced aggregation of chicken erythrocytes were evaluated by hemagglutination inhibition (HI) assay. **e** 293FT cells were transfected with pcDNA3.1 expression plasmids encoding PB2, PB1, PA, NP, and the vNS1-luc plasmid in the absence or presence of PA (3.125, 6.25, 12.5, 25 and 50 μg/ml) or Nucleozin (10 μM). The effect of vRNA transcription was then evaluated by measuring luciferase activity at 48 h post-transfection. Data are expressed as the mean ± SD of three samples in each of three independent experiments. Significance: **p < 0.01 vs. mock control group

Various time-points were assessed to determine the stage(s) at which PA exerted its inhibitory effects in vitro. Briefly, MDCK cells were infected with Vir09 virus (H1N1) (MOI = 1.0) under four different treatment conditions: pre-treatment of viruses, pre-treatment of cells, during adsorption, or after adsorption. At 24 h p.i., the antiviral activity was determined by plaque assay. As shown in Fig. 2a, pretreatment of Vir09 virus with 25 μg/ml PA for 1 h before infection markedly reduced virus titers, suggesting that PA may have direct interaction with IAV particles. However, either the addition of PA during adsorption or pretreatment of cells only weakly inhibited virus multiplication (Fig. 2a), suggesting that PA may not interact with MDCK cells directly. Interestingly, treatment of PA after adsorption also significantly reduced virus titers as compared to the non-treated virus control group (Fig. 2a). Thus, PA may be able to inactivate virus particles directly and block some stages after virus adsorption.

Moreover, another time course study was also performed to explore which viral stage after adsorption is inhibited by PA as described previously [17]. Briefly, Vir09 (MOI = 1.0)-infected MDCK cells were treated with 25 μg/mL of PA for different time intervals, then the virus multiplication at 24 h p.i. was evaluated by plaque assay. The results showed that PA treatment for the first 4 h (0–4 h p.i.) after adsorption resulted in a significant reduction in the virus titer (about 200-fold) (*P* < 0.05) (Fig. 2b). Much less inhibition was noted (less than 20-fold) when PA was added 8 h after infection (> 8 h p.i.), which suggested that PA may be able to inhibit early stages of IAV life cycle (mainly 0–4 h p.i.) (Fig. 2b).

Since PA may be able to inactivate virus particles directly, we then explored whether PA had direct interaction with virus surface NA and HA protein by using the neuraminidase inhibition assay and hemagglutination inhibition (HI) assay. As shown in Fig. 2c, PA could not significantly inhibit the NA activities of Vir09 virus at the concentrations of 12.5–100 μg/ml, while Zanamivir possessed high inhibition percentage (> 80%) at 10 μg/ml, suggesting that PA may have no direct interaction with virus NA protein. Moreover, the results of the HI assay showed that the anti-HA antibodies significantly inhibited the PR8 virus-induced aggregation of chicken erythrocytes at the concentrations of 0.3125–5 μg/mL (Fig. 2d), suggesting that the anti-HA antibody can block the virus attachment to red blood cells through binding to HA. However, PA did not obviously inhibit virus-induced aggregation of chicken erythrocytes even at a concentration of 50 μg/ml (Fig. 2d), suggesting that PA may have no direct interaction with viral HA protein.

Furthermore, we also performed mini-genome assay to evaluate the influence of PA on viral genome replication, which occur during the early stages in viral life cycle. Briefly, 293FT cells were transfected with four expression plasmids encoding PR8 virus PB2, PB1, PA, and NP proteins, and the luciferase-containing plasmid vNS1-luc/pHH21, which encodes a viral-like genome in the absence or presence of PA (3.125, 6.25, 12.5, 25 and 50 μg/ml). The effect of vRNA transcription was then evaluated by measuring luciferase activity at 48 h p.i. The results showed that the positive drug nucleozin caused a notable reduction in luciferase activity at 10 μM as compared with control (DMSO) treatment (Fig. 2e). By contrast, treatment with PA (3.125, 6.25, 12.5, 25 and 50 μg/ml) did not significantly inhibit luciferase activity, suggesting that NP protein may be not the direct target of PA. In summary, virus HA, NA and NP proteins may not be the main targets of PA in vitro.

### The influence of PA on virus mRNA and protein expression

**Fig. 3:**
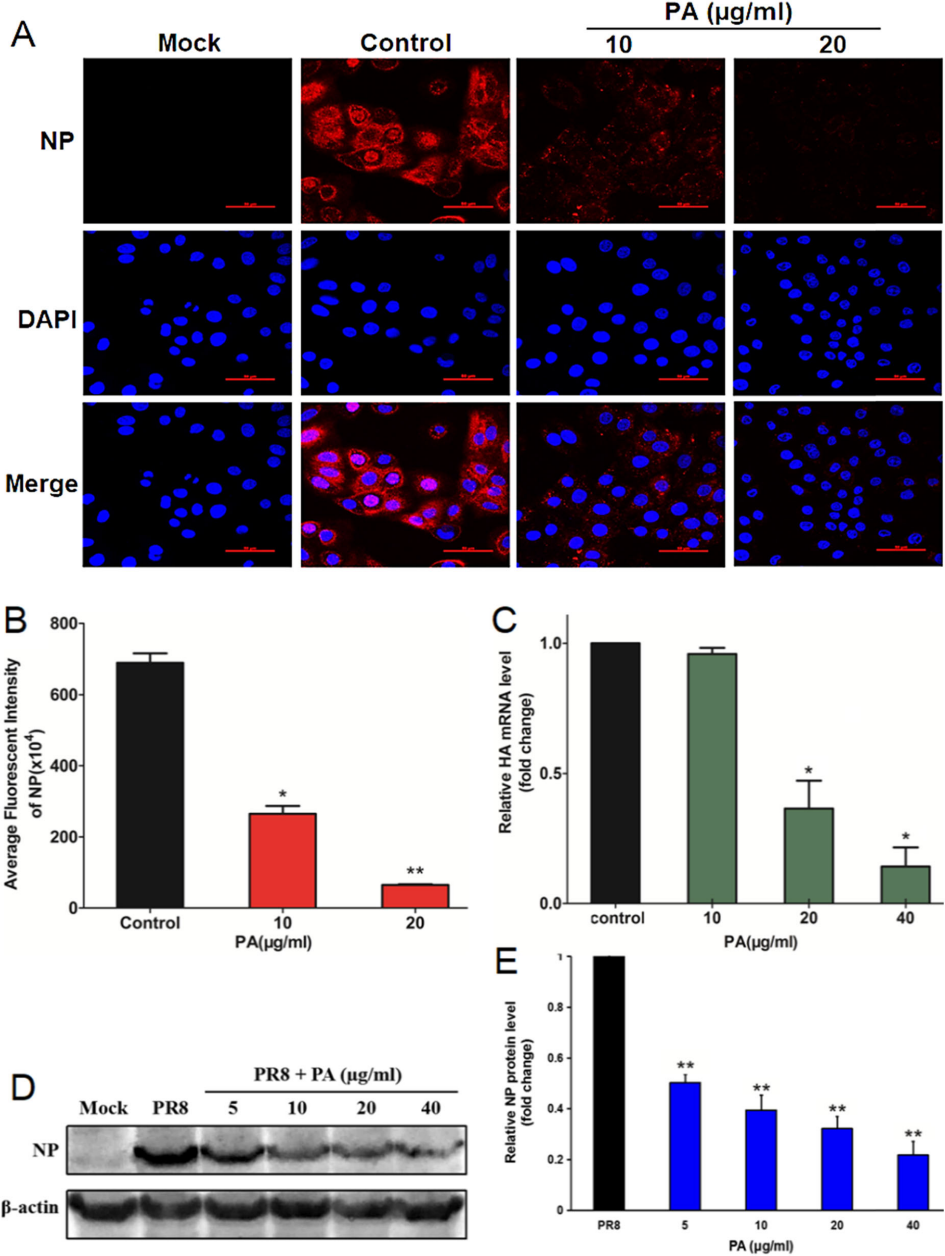
The influence of Patchouli alcohol on virus protein and mRNA expression. **a** Immunofluorescence assay of virus NP protein in H1N1 (Vir09) infected A549 cells at 2 h p.i. Scale bar represents 50 μm. **b** The average fluorescence intensity of NP proteins in (**a**) was measured by ImageJ (NIH) version 1.33u (USA) to calculate the average intensity per unit area of cells of different images (*n* = 30). Significance: ∗*P* < 0.05, ∗∗*P* < 0.01 vs virus control group. **c** Vir09 (MOI = 1.0) infected MDCK cells were treated with different concentrations of PA (10–40 μg/ml), and incubated at 37 °C for 8 h. After that, total RNA was extracted for real-time RT-PCR assay of IAV HA mRNA and cellular β-actin mRNA. The relative amounts of virus HA mRNA were determined using the comparative (^2-ΔΔCT^) method. RNA levels for non-drug treated cells (virus control) were assigned values of 1. Values are means ± SD (*n* = 3). Significance: *P < 0.05 vs. virus control group. **d** MDCK cells were firstly infected with IAV (MOI = 1.0), and then treated with or without PA at indicated concentrations after adsorption. At 8 h p.i., the virus NP protein expression was evaluated by Western blotting. Blots were also probed for β-actin protein as loading controls. **e** Quantification of immunoblot for the ratio of IAV NP protein to actin. The ratio for non-treated virus control group (PR8) were assigned values of 1 and the data presented as mean ± SD (*n* = 3). Significance: **P < 0.01 vs. virus control group (PR8)

Since PA may inhibit some steps after virus adsorption (Fig. 2a and b), the effects of PA on viral protein synthesis and RNA replication were evaluated by using immunofluorescence assay and Real-time RT-PCR assay as described previously [19]. Firstly, Vir09 virus (MOI = 1.0) infected A549 cells were added with 10 or 20 μg/mL of PA after virus adsorption and then incubated at 37 °C for 2 h. After that, viral NP protein expression was detected by immunofluorescence assay. As shown in Fig. 3a, in virus-infected cells without drug treatment, the fluorescence of viral NP proteins could be obviously found in both the cell nucleus and cytoplasm (Fig. 3a), while nearly non fluorescence could be found in the non-infected cells (Fig. 3a). However, after treatment with PA for 2 h, the number of virus antigen-expressing cells was drastically reduced, and only very few fluorescence could be found in the cytoplasm (Fig. 3a). Quantitation of data of the fluorescence intensity in IAV infected cells showed that PA treatment (5, 10 μg/mL) significantly reduced the fluorescence intensity of NP in A549 cells, suggesting that PA may block some steps of IAV life cycle after adsorption to interfering with nuclear import and expression of NP protein (Fig. 3b).

Moreover, the inhibition effect of PA on virus mRNA expression was then evaluated by Real-time RT-PCR assay. IAV (MOI = 1.0) infected cells were added with PA (10, 20, 40 μg/mL) after virus adsorption and then incubated at 37 °C for 8 h. After that, total RNA was extracted for real-time RT-PCR. As shown in Fig. 3c, after treatment with PA (20, 40 μg/mL) for 8 h, the IAV NP mRNA levels decreased to about 36.6 and 14.2% of that of untreated cells after PA treatment, respectively, consistent to the results of immunofluorescence assay.

Furthermore, Western blot assay was also performed to verify the inhibition of PA on viral protein production. MDCK cells were firstly infected with IAV (MOI = 1.0), and then treated with or without PA at indicated concentrations after adsorption. After incubation for 8 h, viral NP protein production was detected by western blot assay. As shown in Fig. 3d and e, the level of viral NP protein was significantly reduced by PA in a dose-dependent manner as compared to that of the non-treated virus control group (PR8) (*P* < 0.01). The treatment with PA at 40 μg/mL reduced the production of IAV NP protein by more than 80% (Fig. 3e). Therefore, PA may also be able to inhibit IAV protein and mRNA expression through interfering with some early steps of virus life cycle.

### The cellular PI3K/Akt and ERK/MAPK signaling pathways may be involved in the anti-IAV actions of PA

**Fig. 4:**
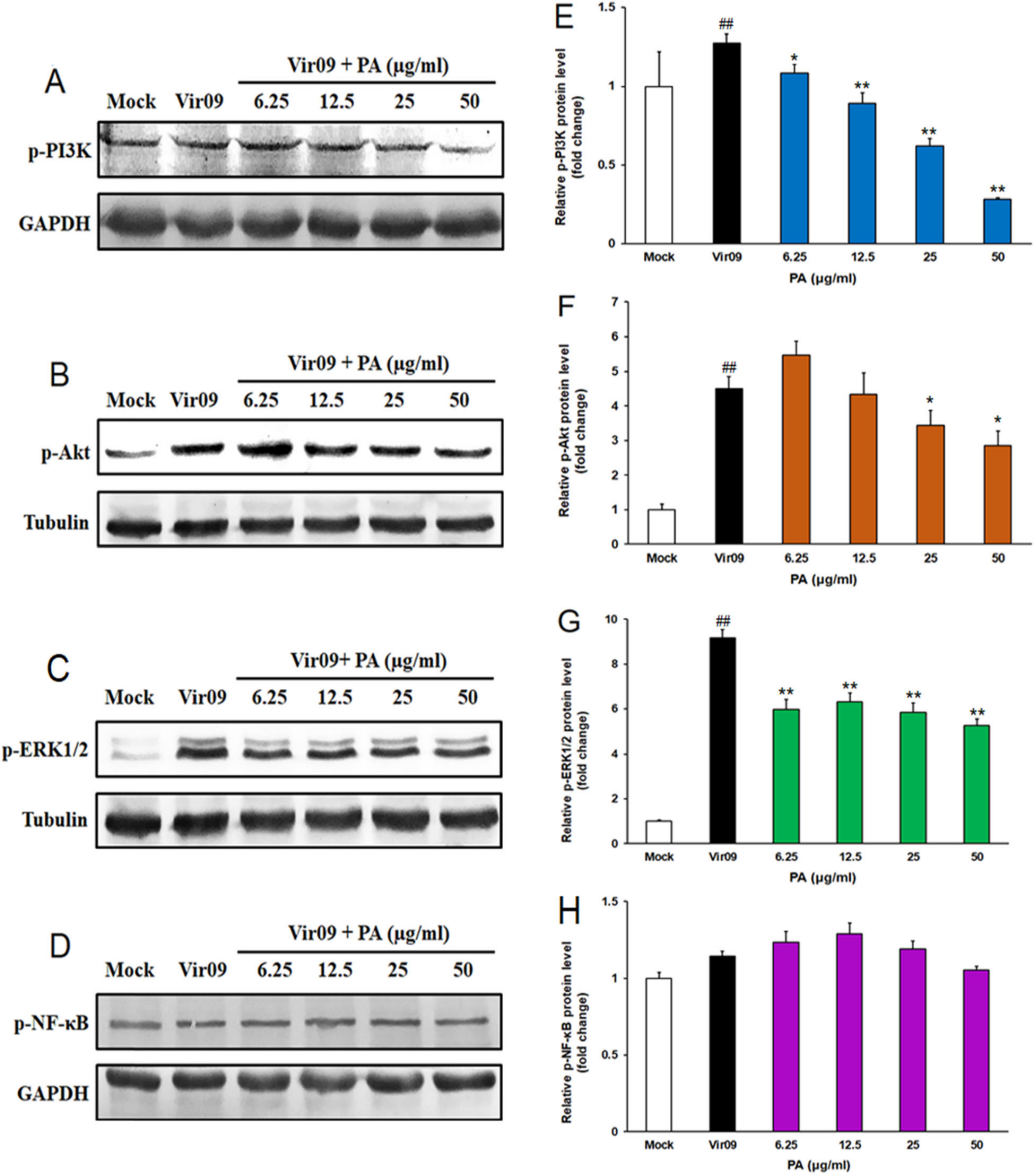
Involvement of PI3K/Akt and ERK/MAPK signaling pathways in the anti-IAV actions of Patchouli alcohol. **a**-**d** Vir09 virus (MOI = 1.0) infected cells were treated with or without PA (6.25, 12.5, 25, 50 μg/ml) for 2 h, and then the phosphorylation of PI3K (**a**), Akt (**b**), ERK1/2 (**c**), and NF-κB (**d**) was evaluated by western blot. Blots were also probed for GAPDH, and α-tubulin proteins as loading controls. The result shown is a representative of three separate experiments. **e**-**h** Quantification of immunoblot for the ratio of p-PI3K (**e**), p-Akt (**f**), p-ERK1/2 (**g**), and p-NF-κB (**h**) protein to GAPDH or tubulin, respectively. The ratio for non-infected cells (M) was assigned values of 1.0 and the data presented as mean ± S.D. (*n* = 3). Significance: ^##^*P* < 0.01 vs. normal control group (Mock); *P < 0.05, **P < 0.01 vs. virus control group (Vir09)

Since PA may inhibit some steps after virus adsorption to reduce IAV mRNA and protein expression in vitro, so we further explored if PA could influence some cellular signaling pathways required for IAV infection. The cellular PI3K/Akt signaling pathway was reported to be required for virus endocytosis and replication, and the inhibitors of PI3K/Akt signaling could inhibit both entry and replication of virus [21, 22]. In this study, after IAV infection for 2 h, the levels of phosphorylated PI3K proteins were significantly increased to about 1.3 fold higher than normal control group in IAV infected cells (*P* < 0.01) (Fig. 4a and e). However, after treatment with PA (6.25, 12.5, 25, 50 μg/ml) for 2 h, the expression level of phosphorylated PI3K significantly decreased from about 1.3 to about 1.1, 0.9, 0.6, and 0.3-fold of normal control group, respectively (*P* < 0.05) (Fig. 4a and e). Moreover, the activation of PI3K can induce the activation of some downstream signals such as Akt, and the level of phosphorylated Akt was truly significantly increased in virus-control group to about 4.5 fold higher than normal control group at 2 h p.i. (*P* < 0.01) (Fig. 4b and f). But treatment with PA (25, 50 μg/ml) for 2 h could significantly reduce the activation of Akt from about 4.5 to about 3.4 and 2.8-fold of normal control group, respectively (Fig. 4b and f). Thus, the PI3K/Akt signaling pathway may be involved in the anti-IAV mechanisms of PA in vitro.

Furthermore, the MAPK signaling pathway was reported to be required for efficient vRNP export from nucleus, and the inhibitors of MAPK pathway could reduce IAV replication and inflammatory symptoms [23–25]. In this study, ERK1/2 protein was significantly activated in virus-control group to approximately 9.2 fold higher than normal control group at 2 h p.i. (*P* < 0.01) (Fig. 4c and g). However, after treatment with PA (6.25, 12.5, 25 and 50 μg/ml) for 2 h, the expression level of phosphorylated ERK1/2 protein significantly decreased from about 9.2 to about 6.0, 6.3, 5.9, and 5.3-fold of normal control group, respectively (*P* < 0.01) (Fig. 4c and g). However, treatment with PA (6.25, 12.5, 25 and 50 μg/ml) for 2 h could not significantly reduce the expression level of phosphorylated NF-κB protein as compared to the virus control group (Fig. 4d and h). Thus, PA may inhibit ERK/MAPK rather than NF-κB pathway to interfere with IAV replication.

**Fig. 5:**
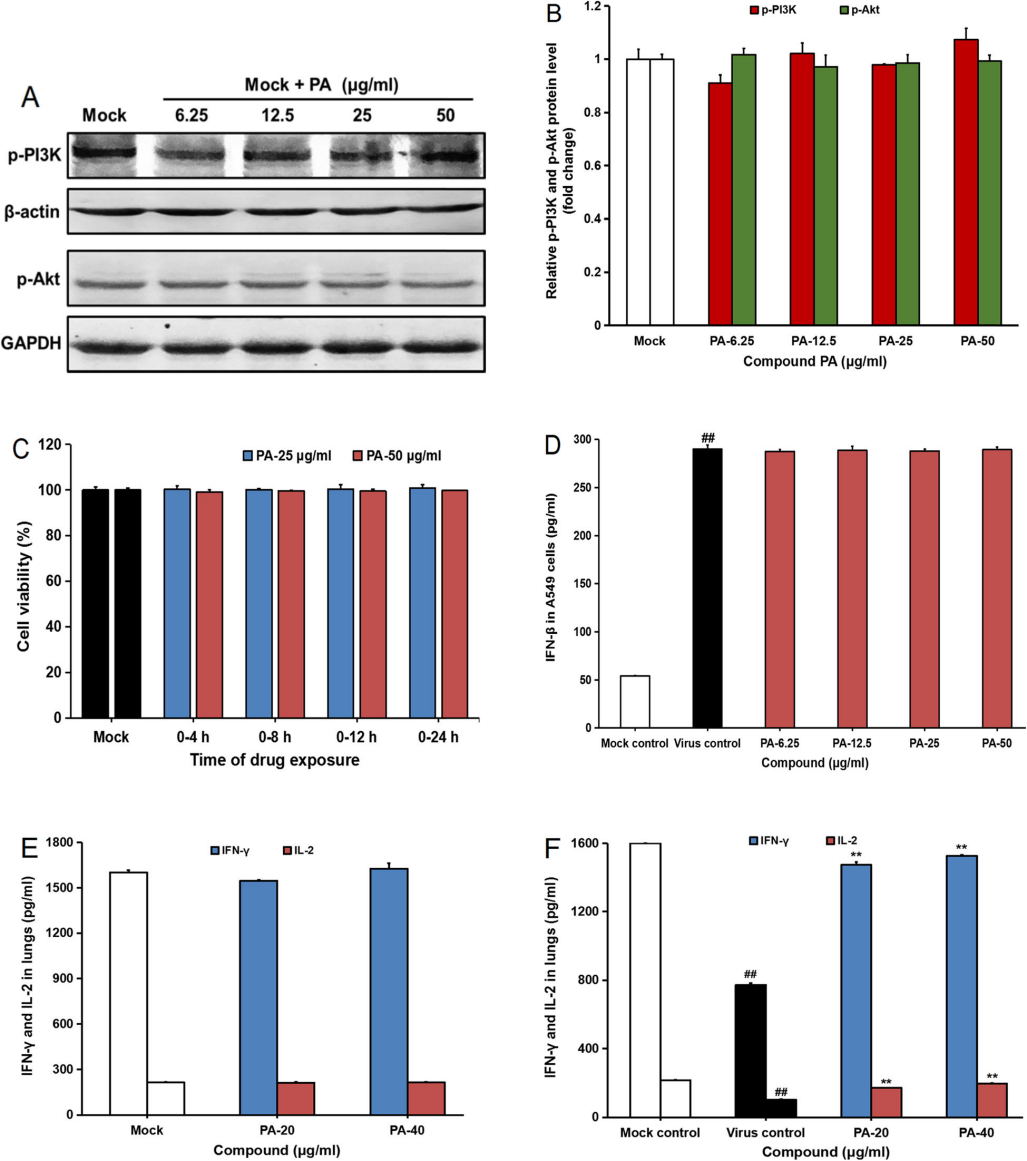
The influence of Patchouli alcohol on host antiviral response. **a** A549 cells were treated with or without PA (6.25, 12.5, 25, 50 μg/ml) for 2 h, and then the phosphorylation of PI3K and Akt proteins was evaluated via western blotting. Blots were also probed for β-actin and GAPDH protein as loading controls. The result shown is a representative of three separate experiments. **b** Plots quantifying the immunoblots (as ratios to β-actin or GAPDH) for p-PI3K and p-Akt proteins, respectively. The ratios for non-treated cells (mock) were assigned values of 1.0 and the data presented as mean ± S.D. (*n* = 3). **c** A549 cells were treated with Patchouli alcohol (50, 25 μg/ml) for specified time period, and then the media were removed and cells were overlaid with compound-free media. Then at 24 h p.i., the cell viability of A549 cells was measured by MTT assay. Values are means ± S.D. (*n* = 3). **d** Vir09 virus (MOI = 0.1) infected cells were treated with or without PA (6.25, 12.5, 25, 50 μg/ml) for 24 h, then the content of IFN-β in the culture supernatants was detected using ELISA kits. Values are means ± S.D. (*n* = 3). ##*P* < 0.01vs. non-infected group (mock control). **e** and **f** After treatment of PA (20 or 40 μg/day) for four days in non-infected mice (**e**) or PR8 virus infected mice (**f**), the production of interferon-γ (IFN-γ) and interleukin 2 (IL-2) in lung tissues was determined by using the ELISA kits for IFN-γ and IL-2. Values are means ± S.D. (*n* = 3). Significance: ##*P* < 0.01 vs. non-infected mock control group; **P < 0.01 vs. virus control group

Moreover, the PI3K/Akt pathway was reported to be associated with host antiviral response [26, 27], so we further explored the influence of PA on immune response by using western blot and ELISA assay. We first evaluate the direct actions of PA on cellular PI3K/Akt pathway in non-infected A549 cells using western blotting. The results showed that PA treatment (6.25, 12.5, 25, 50 μg/ml) could not significantly influence the activation of PI3K and Akt proteins in the non-infected A549 cells (Fig. 5a and b), suggesting that the inhibition of PI3K/Akt pathway by PA may be related to its inhibition of IAV infection. Treatment of PA for different time intervals within 24 h showed no significant cytotoxicity to non-infected A549 cells (Fig. 5c). In addition, IAV infection significantly increased the production of cellular interferon-β (IFN- β) in Vir09 virus infected A549 cells, however, PA treatment (6.25, 12.5, 25, 50 μg/ml) could not significantly influence the production of IFN-β as compared to the virus control group (Fig. 5d), suggesting that PA had no direct action on cellular antiviral response. Furthermore, we also evaluated the influence of PA on the production of interferon-γ (IFN-γ) and interleukin 2 (IL-2) in mice with or without PR8 virus infection. As shown in Fig. 5e, intranasal treatment of PA (20 or 40 μg/day) for four days had no significant influence on the production of IFN-γ and IL-2 in non-infected mice. However, PA treatment could significantly reverse the reduction of IFN-γ and IL-2 in IAV infected mice (Fig. 5f), suggesting that the enhancement of PA on type-II interferon system may be related to its inhibition of IAV inhibition in vivo. Thus, the inhibition of PI3K/Akt pathway by PA may be related to its inhibition of IAV infection rather than direct actions on host antiviral response.

### Intranasal PA application supports survival of mice infected with IAV

**Fig. 6:**
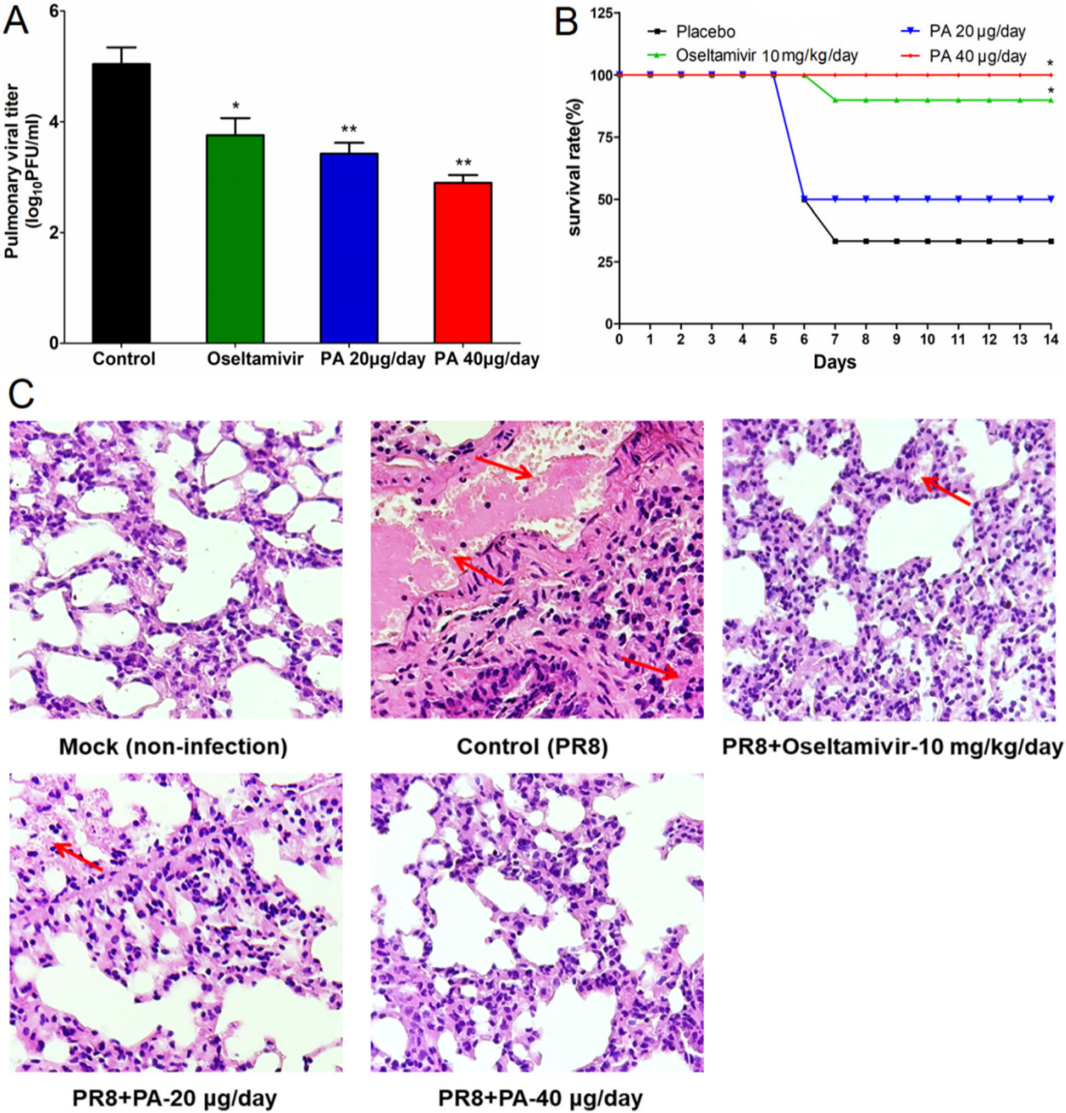
The anti-IAV effects of Patchouli alcohol in vivo. **a** Viral titers in lungs. After treatment with Oseltamivir (10 mg/kg/day) or PA (20 or 40 μg/day) for 4 days, the pulmonary viral titers were evaluated by plaque assay. Values are the mean ± SD (*n* = 3). Significance: *P < 0.05, **P < 0.01 vs virus control group. **b** Survival rate. IAV infected mice received therapy with Oseltamivir (10 mg/kg/day) or PA (20 or 40 μg/day) for the entire experiment. Results are expressed as percentage of survival, evaluated daily for 14 days. Significance: *P < 0.05 vs. virus control group (placebo). **c** Histopathologic analyses of lung tissues on Day 4 p.i. by HE staining (× 10). The representative micrographs from each group were shown (*n* = 5 mice/group). Mock: non-infected lungs; Control: IAV infected lungs without drugs; Oseltamivir: IAV infected lungs with Oseltamivir (10 mg/kg/day) treatment; PA 20 μg/day: IAV infected lungs with PA (20 μg/day) treatment; PA 40 μg/day: IAV infected lungs with PA (40 μg/day) treatment. The red arrows indicate the presence of inflammatory cells in the alveolar walls and serocellular exudates in the lumen

The anti-IAV effects of PA in vivo were further explored using a mouse pneumonia model [18]. In brief, IAV-infected mice received intranasal administration of PA (20 or 40 μg/day) or placebo (PBS) once daily for the entire experiment, and the selected subset of treated, infected mice were then sacrificed on Day 4 and the tissue samples were removed for further analysis. Subsequently, the pulmonary viral titers were determined by plaque assay [22]. As shown in Fig. 6a, after treatment of PA (20, 40 μg/day) for 4 days, the pulmonary viral titers significantly decreased compared to that of the virus control group (*P* < 0.01), suggesting that intranasal therapy with PA could inhibit IAV multiplication in mice lungs. Oral therapy of oseltamivir (10 mg/kg/day) also showed significant reduction of virus titers in mice lungs (*P* < 0.05) (Fig. 6a).

Moreover, the survival experiments were also performed to evaluate the effects of PA on the survival of IAV-infected mice. As shown in Fig. 6b, intranasal administration with PA (40 μg/day) significantly increased survival rates as compared to the placebo-treated control group (*P* < 0.05). By day 14 post infection, only 30% of the individuals in the placebo group survived whereas 100% of animals in the PA (40 μg/day)-treated group survived, superior to that in Oseltamivir (10 mg/kg/day)-treated group (90%). PA treatment at 20 μg/day also increased the survival rate of IAV infected mice (50%) but without significance (Fig. 6b).

To further evaluate the effects of PA on viral pneumonia in mice, histopathology analysis was also performed as described previously [18]. As shown in Fig. 6c, lung tissues in virus-control group showed marked infiltration of inflammatory cells in the alveolar walls and the presence of massive serocellular exudates in the lumen. However, after treatment with PA (20 or 40 μg/day) for 7 days, the lung tissues showed intact columnar epithelium in the bronchiole even in the presence of some serocellular exudates in the lumen (Fig. 6c). Mice treated with Oseltamivir (10 mg/kg/day) also had intact columnar epithelium (Fig. 6c). Thus, PA may be able to attenuate pneumonia symptoms in IAV infected mice.

## Discussion

Natural products from Chinese medicine have been attracting more and more attention of pharmacists. Patchouli alcohol (PA), a tricyclic sesquiterpene extracted from *Pogostemonis Herba*, was reported to possess anti-viral activities against different viruses especially influenza virus [14–16]. In the current study, we found that PA could inhibit different influenza A virus replication in vitro, and the pandemic H1N1 virus (Vir09) was most susceptible to PA treatment (IC_50_ < 6.5 μg/ml). Intranasal administration of PA significantly promoted the survival rate of mice and attenuated pneumonia symptoms in IAV infected mice, comparable to the positive control drug Oseltamivir. Thus, Patchouli alcohol merits further investigation as a novel anti-IAV agent in the future.

The time-of-addition assay indicated that pretreatment of IAV with PA before infection or addition of PA during adsorption markedly reduced virus multiplication (Fig. 2), suggesting that PA may have direct inactivation effects on IAV particles. PA was reported to inhibit H2N2 virus replication mainly through inhibition of the functions of virus neuraminidase [16]. However, in contrast to the previous studies, we found that PA could not significantly inhibit the NA activity of H1N1 virus, and did not significantly block HA mediated aggregation of chicken red blood cells. Thus, PA may be not able to directly bind to virus surface proteins but may interfere with the interaction between IAV and cell receptors. Interestingly, post-treatment of cells with PA after adsorption also dramatically inhibited virus multiplication, suggesting that PA may also block some stages after virus adsorption.

Cellular PI3K/Akt signaling pathway is known to be able to augment replication of several viruses, and may be associated with lytic infections of both RNA and DNA viruses, including influenza A virus [21]. Some inhibitors of PI3K or its downstream signal Akt could significantly block virus entry and replication [20–22]. Herein, Patchouli alcohol was found to be able to significantly inhibit the phosphorylation of PI3K and Akt proteins in IAV-infected cells (Fig. 5), suggesting that PA may inhibit the activation of PI3K/Akt signaling pathway to block virus infection and replication. However, PA could not influence the activation of PI3K/Akt pathway in non-infected A549 cells and could not directly enhance the interferon system in vitro, suggesting that the inhibition of PI3K/Akt pathway by PA may be related to its inhibition of IAV infection rather than direct actions on host antiviral response. Moreover, the MAPK and NF-κB signaling pathways were reported to be required for efficient vRNP export from nucleus and virus RNA synthesis, and the inhibitors of MAPK pathway could reduce both IAV replication and inflammatory symptoms [23–25]. In this study, PA significantly reduced the activation of ERK1/2 rather than NF-κB in IAV infected cells, suggesting that ERK/MAPK rather than NF-κB pathway may be involved in the anti-IAV actions of PA. Considered that PA could significantly inhibit virus mRNA and protein expression in IAV infected cells, we posit that PA may interfere with the activation of PI3K/Akt and ERK/MAPK signaling pathways, thus inhibiting the invasion and subsequent replication of IAV.

The in vivo anti-IAV effects of PA were also explored in a murine pneumonia model of influenza. Intranasal treatment of PR8-infected mice with PA markedly improved their survival and decreased the pulmonary virus titers (Fig. 6). Moreover, the histopathological analysis indicated that PA treatment also attenuated the pneumonia symptoms in IAV-infected lungs, comparable to the effects of Oseltamivir. However, PA treatment exerted obvious therapeutic effect only when starting earlier (4 h p.i.), which will restrict its clinical application to some extent. Different to the oral administration of Oseltamivir, PA was administrated through intranasal, and low dose therapy of PA (20 μg/day) had comparable anti-IAV effects to Oseltamivir (10 mg/kg/day), suggesting that PA may be used alone or combined with Oseltamivir for treatment of influenza by different administration.

## Conclusions

In summary, PA possesses anti-IAV activities both in vitro and in vivo, and may block IAV infection through targeting virus particles and cellular PI3K/Akt and ERK/MAPK signaling pathways. Although further studies of the antiviral effects of PA against other IAV strains (H3N2 or H5N1) will be required to advance it for drug development, PA has the potential to be developed into a novel nasal drop for influenza therapy and prophylaxis in the future.

## Data Availability

The datasets used during the current study are available from the corresponding author on reasonable request.

## Abbreviations

BCIP: 5-bromo-4-chloro-3-indolyl phosphate toluidine
CC_50_: 50% cytotoxicity concentration
cRBC: Chicken red blood cell;
HA: Hemagglutin
HI: Hemagglutination inhibition
IAV: Influenza virus
MDCK: Madin-Darby canine kidney
MOI: Multiplicity of infection
NBT: P-nitro blue tetrazolium chloride
PA: Patchouli alcohol
SD: Standard deviations
TBS: Tris-buffered saline

## References

[1] Palese P, Shaw ML (2007). Orthomyxoviridae . the viruses and their replication. Fields virology.

[2] Medina RA (2018). 1918 influenza virus . 100 years on, are we prepared against the next influenza pandemic?. Nat Rev Microbiol.

[3] Kadam RU, Wilson IA (2017). Structural basis of influenza virus fusion inhibition by the antiviral drug Arbidol. Proc Natl Acad Sci U S A.

[4] Takamatsu K, Marumo S, Fukui M, Hata A (2017). Safety and efficacy of anti-influenza drugs, intravenous peramivir against influenza virus infection in elderly patients with underlying disease. J Microbiol Immunol Infect.

[5] Schirmer P, Holodniy M (2009). Oseltamivir for treatment and prophylaxis of influenza infection. Expert Opin Drug Saf.

[6] Hussain M, Galvin HD, Haw TY, Nutsford AN, Husain M (2017). Drug resistance in influenza A virus: the epidemiology and management. Infect Drug Resist.

[7] Moscona A (2009). Global transmission of oseltamivir-resistant influenza. N Engl J Med.

[8] Mc Mahon A, Martin-Loeches I (2017). The pharmacological management of severe influenza infection - 'existing and emerging therapies'. Expert Rev Clin Pharmacol.

[9] Hu G, Peng C, Xie X, Zhang S, Cao X (2017). Availability, pharmaceutics, security, pharmacokinetics, and pharmacological activities of patchouli alcohol. Evid Based Complement Alternat Med.

[10] Yang Y, Kinoshita K, Koyama K, Takahashi K, Tai T, Nunoura Y, Watanabe K (1999). Anti-emetic principles of Pogostemon cablin (Blanco) Benth. Phytomedicine..

[11] Lu TC, Liao JC, Huang TH, Lin YC, Liu CY, Chiu YJ, Peng WH (2011). Analgesic and anti-inflammatory activities of the methanol extract from *Pogostemon cablin*. Evid Based Complement Alternat Med.

[12] Yu XD, Xie JH, Wang YH, Li YC, Mo ZZ, Zheng YF, Su JY, Liang YE, Liang JZ, Su ZR, Huang P (2015). Selective antibacterial activity of patchouli alcohol against helicobacter pylori based on inhibition of urease. Phytother Res.

[13] Kiyohara H, Ichino C, Kawamura Y, Nagai T, Sato N, Yamada H (2012). Patchouli alcohol: in vitro direct anti-influenza virus sesquiterpene in Pogostemon cablin Benth. J Nat Med.

[14] Li YC, Peng SZ, Chen HM, Zhang FX, Xu PP, Xie JH, He JJ, Chen JN, Lai XP, Su ZR (2012). Oral administration of patchouli alcohol isolated from Pogostemonis Herba augments protection against influenza viral infection in mice. Int Immunopharmacol.

[15] Wu H, Li B, Wang X, Jin M, Wang G (2011). Inhibitory effect and possible mechanism of action of patchouli alcohol against influenza A (H2N2) virus. Molecules..

[16] Wang W, Zhang P, Hao C, Zhang XE, Cui ZQ, Guan HS (2011). In vitro inhibitory effect of carrageenan oligosaccharide on influenza A H1N1 virus. Antivir Res.

[17] Wang W, Wu J, Zhang X, Hao C, Zhao X, Jiao G, Shan X, Tai W, Yu G (2017). Inhibition of influenza A virus infection by fucoidan targeting viral neuraminidase and cellular EGFR pathway. Sci Rep.

[18] Livak KJ, Schmittgen TD (2001). Analysis of relative gene expression data using real-time quantitative PCR and the 2(−Delta Delta C(T)) method. Methods.

[19] Fukushi M, Ito T, Oka T, Kitazawa T, Miyoshi-Akiyama T, Kirikae T, Yamashita M, Kudo K (2011). Serial histopathological examination of the lungs of mice infected with influenza A virus PR8 strain. PLoS One.

[20] Wang W, Yin R, Zhang M, Yu R, Hao C, Zhang L, Jiang T (2017). Boronic acid modifications enhance the anti-influenza A virus activities of novel quindoline derivatives. J Med Chem.

[21] Kindrachuk J, Ork B, Hart BJ, Mazur S, Holbrook MR, Frieman MB, Traynor D, Johnson RF, Dyall J, Kuhn JH (2015). Antiviral potential of ERK/MAPK and PI3K/AKT/mTOR signaling modulation for Middle East respiratory syndrome coronavirus infection as identified by temporal kinome analysis. Antimicrob Agents Chemother.

[22] Pleschka S, Wolff T, Ehrhardt C, Hobom G, Planz O, Rapp UR, Ludwig S (2001). Influenza virus propagation is impaired by inhibition of the Raf/MEK/ERK signalling cascade. Nat Cell Biol.

[23] Kumar N, Liang Y, Parslow TG, Liang Y (2011). Receptor tyrosine kinase inhibitors block multiple steps of influenza A virus replication. J Virol.

[24] Pinto R, Herold S, Cakarova L, Hoegner K, Lohmeyer J, Planz O, Pleschka S (2011). Inhibition of influenza virus-induced NF-kappaB and Raf/MEK/ERK activation can reduce both virus titers and cytokine expression simultaneously in vitro and in vivo. Antivir Res.

[25] Ehrhardt C, Ludwig S (2009). A new player in a deadly game: influenza viruses and the PI3K/Akt signalling pathway. Cell Microbiol.

[26] Oh C, Ryoo J, Park K, Kim B, Daly MB, Cho D, Ahn K (2018). A central role for PI3K-AKT signaling pathway in linking SAMHD1-deficiency to the type I interferon signature. Sci Rep.

[27] Barnard DL (2009). Animal models for the study of influenza pathogenesis and therapy. Antivir Res.

